# ARHGAP39 is a prognostic biomarker involved in immune infiltration in breast cancer

**DOI:** 10.1186/s12885-023-10904-4

**Published:** 2023-05-15

**Authors:** Litong Yao, Yuwei Li, Siyuan Li, Mozhi Wang, Hongyi Cao, Ling Xu, Yingying Xu

**Affiliations:** 1 Department of Breast Surgery, the First Hospital of China Medical University, Shenyang, 110001 Liaoning China; 2 Department of Breast Surgery, Fudan University Shanghai Cancer Center, Shanghai, China; 3 Department of Pathology, the First Hospital of China Medical University and College of Basic Medical Sciences, Shenyang, Liaoning, China; 4 Department of Medical Oncology, the First Hospital of China Medical University, Shenyang, China

**Keywords:** ARHGAP39, Breast cancer, Biomarker, Immune infiltration, Prognosis

## Abstract

**Background:**

Current studies on the role of ARHGAP39 mainly focused on its effect on neurodevelopment. However, there are few studies on the comprehensive analysis of ARHGAP39 in breast cancer.

**Methods:**

ARHGAP39 expression level was analyzed based on the Cancer Genome Atlas (TCGA), the Genotype-Tissue Expression Project (GTEx), and the Clinical Proteomic Tumor Analysis Consortium (CPTAC) database and validated by qPCR in various cell lines and tumor tissues. The prognostic value was analyzed using Kaplan–Meier curve analysis. CCK-8 and transwell assays were conducted to identify the biological function of ARHGAP39 in tumorigenesis. Signaling pathways related to ARHGAP39 expression were identified by the GO and KEGG enrichment analysis and gene set enrichment analysis (GSEA). The correlations between ARHGAP39 and cancer immune infiltrates were investigated via TIMER, CIBERSORT, ESTIMATE and tumor-immune system interactions database (TISIDB).

**Results:**

ARHGAP39 was overexpressed in breast cancer and associated with poor survival outcomes. In vitro experiments revealed that ARHGAP39 could facilitate the proliferation, migration, and invasion capability of breast cancer cells. GSEA analysis showed that the main enrichment pathways of ARHGAP39 was immunity-related pathways. Considering the immune infiltration level, ARHGAP39 was negatively associated with infiltrating levels of CD8 + T cell and macrophage, and positively associated with CD4 + T cell. Furthermore, ARHGAP39 was significantly negatively correlated with immune score, stromal score, and ESTIMATE score.

**Conclusions:**

Our findings suggested that ARHGAP39 can be used as a potential therapeutic target and prognostic biomarker in breast cancer. ARHGAP39 was indeed a determinant factor of immune infiltration.

**Supplementary Information:**

The online version contains supplementary material available at 10.1186/s12885-023-10904-4.

## Introduction

Breast cancer is the most common cancer in women and the main cause of cancer‐related mortality [1]. The typical treatment options for patients with breast cancer include surgery, chemotherapy, radiotherapy, and endocrine therapy which can significantly improve clinical efficacy and survival outcomes [2, 3]. Despite improved diagnostic techniques and therapeutic strategies for breast cancer, patients still suffer from disease relapse and metastasis, and have a poor prognosis [4, 5]. The tumor microenvironment (TME) contains a combination of tumor, immune and stromal cell components. Increasing evidence confirm that the TME represent a milieu that enable tumor cells to acquire and maintain the hallmarks of cancer [6]. The crosstalk among tumor cells and tumor-infiltrating immune cells within the TME, for example, B cells, T cells and macrophages, shaping a tumor immune microenvironment can positively or negatively regulate the induction of efficient antitumor immunity [7]. Research into the impact of tumor-infiltrating immune cells on breast cancer development could help facilitate more effective immunotherapies for patients and require further investigation. In addition, the identification of suitable and novel biomarkers for tumor diagnosis, management and prognosis based on the underlying molecular mechanisms of breast cancer is in urgent need [8–11].

A member of the Ras superfamily, the Rho GTPase family, which can serve as a key regulator of a diverse array of biological processes including cytoskeleton dynamic remodeling and assembling, vesicle trafficking, cell polarity regulation, cell transcriptional control, cell proliferation, adhesion, migration, and invasion [12–14]. Rho GTPase catalyzes the conversion between the active GTP-bound form and inactive GDP-bound form of Ras superfamily [15]. An ARHGAP family gene encoding GTPase-activating protein can negatively regulate Rho GTPases. Members of ARHGAP family have been reported to engage in tumorigenesis of multiple cancer types, and previous studies have elucidated that the ARHGAP family can influence immune infiltration and regulate immune microenvironment [16–20]. While specific ARHGAP genes with prognostic value and biological function have also been characterized in breast cancer, several members of ARHGAPs have limited evidence for the role in cancer. The functional roles of ARHGAP33 (TCGAP), ARHGAP47 (TAGAP), ARHGAP11B, ARHGAP39 in tumorigenesis remain uncertain [21]. Because of transcriptomic and proteomic analysis of above molecules, we focused on the biological functions ARHGAP39 on the development of breast cancer.

In current study, we conducted a comprehensive bioinformatical analysis using multiple databases to determine the diagnostic and prognostic values of ARHGAP39. The transcriptional expression of ARHGAP39 in tissues and various cell lines was detected by quantitative real-time PCR (qRT-PCR). In vitro experiments including cell viability, colony formation, transwell and wound healing assays were used to measure its ability of proliferation, migration, and invasion. Mechanistically, we constructed a co-expression network of ARHGAP39 and identified related signaling pathways by enrichment analysis. Moreover, we explored the impact of ARHGAP39 in regulating immune infiltration of breast cancer. Our findings provide insight into the fundamental role of ARHGAP39 in breast cancer and propose underlying mechanisms of ARHGAP39 in immune infiltrates.

## Methods

### Data sources and database

ARHGAP39 gene expression profiles derived from the Cancer Genome Atlas (TCGA) and the Genotype-Tissue Expression Project (GTEx) were obtained from the UCSC XENA (https://xena.ucsc.edu/). Data for ARHGAP39 protein expression and clinical information were obtained from the Clinical Proteomic Tumor Analysis Consortium (CPTAC) database (https://proteomics.cancer.gov/programs/cptac/) [22]. The relationship between ARHGAP39 and clinicopathological features was analyzed at both mRNA and protein levels using ULCAN online tools (http://ualcan.path.uab.edu/) [23]. The GSE1456 dataset was downloaded from the Gene Expression Omnibus (GEO) dataset and used for verification of the relapse free survival (RFS), overall survival (OS), and disease-specific survival (DSS) of ARHGAP39 through Kaplan–Meier curve analysis [24, 25]. We performed survival analysis for DNA microarray and METABRIC using Breast Cancer Gene-Expression Miner v4.9 (bc-GenExMiner v4.9). The protein-protein interactions (PPI) network of ARHGAP39 was conducted by STRING database (https://string-db.org/cgi/input.pl). Human Protein Atlas (HPA) database (http://www.proteinatlas.org/), was applied to display ARHGAP39 expression level in various cell lines for a multidimensional exploration and to explore subcellular locations in breast cancer cells [26]. The antibody used for immunofluorescence was HPA044491.

### Analysis of genes co-expressed with ARHGAP39

LinkedOmics (http://www.linkedomics.org) was used to analyze co-expressed genes in correlation with ARHAGAP39 [27]. Pearson’s correlation coefficient was used to measure statistical correlation. The visualization of resulting plots was displayed by R’s ggplot2 software package. Heatmaps were generated for top 50 genes positively and negatively associated with ARHGAP39 expression. The grouping basis of the heatmap was the median expression of ARHGAP39.

### Enrichment analysis

Gene Ontology (GO) and Kyoto Encyclopedia of Genes and Genomes (KEGG) pathway enrichment analyses were carried out by the R package’s cluster Profiler program [28, 29]. The biological process (BP), cellular component (CC), molecular function (MF), and KEGG pathways among ARHGAP39 co-expressed genes were visualized by ggplot2 software package. A false discovery rate (FDR) > 2.5, and *p *< 0.05 were considered significant.

### Gene Set Enrichment Analysis

Gene Set Enrichment Analysis (GSEA) analysis was performed by GSEA (www.gsea-msigdb.org/gsea/index.jsp) [30]. Patients with invasive breast cancer from the TCGA BRCA dataset were divided into two groups based on median ARHGAP39 mRNA level to determine crucial biological pathways and the underlying mechanism of ARHGAP39.

An FDR < 0.25, *p* < 0.05, and normalized enrichment score (|NES|) > 1 were considered significant.

### Tumor immune infiltrating cells and the tumor microenvironment

The relationship between tumor immune-infiltrating cells (B cells, CD4 + T cells, CD8 + T cells, neutrophils, macrophages, and dendritic cells) and ARHGAP39 expression level in breast cancer samples was analyzed by the TIMER database (https://cistrome.shinyapps.io/timer/) [31]. Moreover, we examined the association between ARHGAP39 somatic copy number variation (CNV) and immune infiltrates in the somatic copy number alteration (SCNA) module. The relationship between survival outcome and abundance of ARHGAP39 expression and immune cells was evaluated by Kaplan–Meier analysis. Additionally, the interaction between ARHGAP39 and immune cell markers was explored in TIMER database. The proportions of 22 immune cell types were calculated using the R’s CIBERSORT software package, and the Pearson's analysis was used to evaluate the correlation between proportions and ARHGAP39 level [32]. The stromal score, immune score, and Estimate score representing the abundance of different components within the tumor microenvironment were obtained using the R’s ESTIMATE software package and Pearson's analysis [33]. Furthermore, the association between ARHGAP39 expression level and immunomodulators was performed by TISIDB database (http://cis.hku.hk/TISIDB/index.php) and Spearman's analysis to determine the potential immunomodulatory mechanism [34].

### Patient samples

Breast cancer tissues and adjacent normal tissues were collected from the First Hospital of China Medical University. Samples were cryopreserved in liquid nitrogen prior to RNA extraction. The protocol of this study was approved by the Ethics Committee of the First Hospital of China Medical University (Approval number: AF- SOP-07–1.1–01). Written informed consent was obtained from all participants. All methods in the study followed relevant guidelines & regulations.

### Cell culture

The human breast cancer cell lines CAL51, MDA-MB-231, MCF7, SUM159PT, and HCC1806 were cultured in DMEM medium, and BT549 was cultured in RPMI 1640 medium supplemented with 10% fetal bovine serum (FBS), and 1% penicillin- streptomycin. MCF10A was cultured in human mammary epithelial cell growth complete medium. Cell lines were cultured in a humidified environment at 37 °C comprising 95% air and 5% CO2.

### Small interfering RNA transfection

Small interfering RNA (siRNA) was synthesized by GenePharma. The sequences of siRNAs were as follows: siARHGAP39-1: GAAAGAAACCCAAGCCUUATT and siARHGAP39-2: CACCAGGAGUGUUCCUUGATT. siRNA transfection was conducted with Hieff Trans™ liposomal transfection reagent (Yeasen) based on the manufacturer’s protocol.

### Quantitative RT-PCR analysis

Total RNA was extracted using RNAiso Plus (Takara) according to the manufacturer’s protocol. Total RNA was reversely transcribed into cDNA using the PrimeScript RT reagent Kit (TaKaRa). qRT-PCR was performed using SYBR Premix Ex Taq (Takara). The differential expression of ARHGAP39 in cell lines and tissues was measured by qRT-PCR. ARHGAP39 expression level was estimated using the 2^−ΔΔCt^ method and normalized to GAPDH expression. The primers sequences used for qRT-PCR were as follows:

ARHGAP39-F 5′-ATGTCCCAGACGCAGGACTA-3′

ARHGAP39-R 5′-CGCGGTTCGATGATCTCCA-3′

GAPDH-F 5′-GGAGCGAGATCCCTCCAAAAT-3′

GAPDH-R 5′-GGCTGTTGTCATACTTCT CATGG-3′.

### Cell viability assay

Cell Counting Kit-8 (CCK-8) (Yeasen) was used for the determination of cell viability following the manufacturer’s protocol. CAL51 and MDA-MB-231 cells were planted at a density of 2 × 10^3^ in 96-well plates after transfection for 12 h. The absorbance values at a wavelength of 450 nm were measured in a Microplate Reader. The absorbance at each time point for four days was used to plot the cell proliferation curve.

### Transwell migration and invasion assays

Transwell migration assays were carried out with Transwell chambers and transwell invasion assays were conducted using Matrigel (BD Biosciences) inserts in a 24-well plate. A total of 4 × 10^4^ cells were planted into upper chambers with no-serum medium at 12 h post transfection. Medium containing 20% FBS was added into the lower chambers. Cells on the top of inserts were scraped off, and migrated and invaded cells on lower surface were fixed with methanol and stained with 0.1% crystal violet following incubation for 24–36 h at 37 °C. The numbers of migrating and invading cells were counted in randomly selected microscope fields and averaged.

### Statistical analysis

Statistical analyses were carried out using GraphPad (version 9.4.1), R software (version 4.0.3) and online tools. The survival curves were generated by the Kaplan–Meier plot method and analyzed by the log-rank test. Correlation coefficients were calculated using the Pearson or Spearman test. Data were presented as the mean ± standard deviation (SD) from at least three independent experiments. *P* < 0.05 was considered as statistically significant (*, *p* < 0.05; **, *p* < 0.01; ***, *p* < 0.001; ns, no significance).

## Results

### ARHGAP39 expression analysis in pan-cancer

**Fig. 1:**
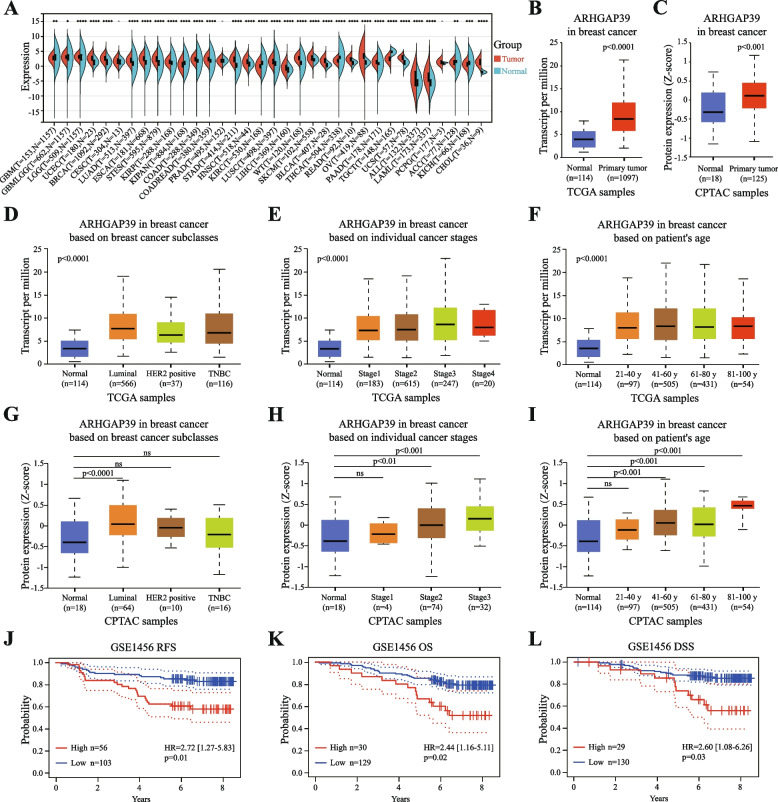
ARHGAP39 is overexpressed in breast cancer and associated with poor prognosis. **A** ARHGAP39 mRNA expression in pan-cancer based on the TCGA database. **B** Difference in ARHGAP39 mRNA expression level between breast cancer tissues and normal tissues in TCGA database. **C** Difference in ARHGAP39 protein expression between breast cancer and normal tissues in CPTAC database. **D**-**F** The correlation between ARHGAP39 mRNA expression level and molecular subtypes, pathological stages, and age. **G**-**I** The correlation between ARHGAP39 protein expression level and molecular subtypes, pathological stages, and age. (J-L) Kaplan–Meier plotter of the relapse free survival (RFS), overall survival (OS), and disease-specific survival (DSS) based on ARHGAP39 expression level of GSE1456

We analyzed the differences in ARHGAP39 mRNA expression between tumor and its adjacent normal tissues in pan-cancer using the TCGA and GTEx datasets (Fig. 1A). Compared with normal tissues, ARHGAP39 was overexpressed in 24 cancers: breast invasive carcinoma (BRCA), glioma (GBMLGG), brain lower grade glioma (LGG), uterine corpus endometrial carcinoma (UCEC), lung adenocarcinoma (LUAD), esophageal carcinoma (ESCA), stomach and esophageal carcinoma (STES), kidney renal papillary cell carcinoma (KIRP), colon adenocarcinoma (COAD), colon adenocarcinoma/rectum adenocarcinoma esophageal carcinoma (COADREAD), stomach adenocarcinoma (STAD), head and neck squamous cell carcinoma (HNSC), lung squamous cell carcinoma (LUSC), liver hepatocellular carcinoma (LIHC), high-risk Wilms tumor (WT), skin cutaneous melanoma (SKCM), bladder urothelial carcinoma (BLCA), rectum adenocarcinoma (READ), ovarian cancer (OV), pancreatic adenocarcinoma (PAAD), uterine carcinosarcoma (UCS), acute lymphoblastic leukemia (ALL), acute myeloid leukemia (LAML), and cholangiocarcinoma (CHOL). ARHGAP39 was significantly decreased in 7 cancers: glioblastoma multiforme (GBM), pan-kidney cohort (KIPAN), kidney renal clear cell carcinoma (KIRC), thyroid carcinoma (THCA), testicular germ cell tumors (TGCT), adrenocortical carcinoma (ACC), and kidney chromophobe (KICH).

### ARHGAP39 expression analysis in breast cancer

We analyzed mRNA and protein expression of ARHGAP39 in human breast cancer based on TCGA and CPTAC databases. The mRNA level was overexpressed in tumor tissues compared to normal tissues (Fig. 1B). Consistently, ARHGAP39 protein expression was upregulated in breast cancer samples compared with normal samples (Fig. 1C). Next, we studied mRNA expression level of ARHGAP39 in different molecular subtypes, cancer stages, and ages. ARHGAP39 expression in patients with luminal breast cancer was higher than that in HER2-positive breast cancer (Fig. 1D, *p* < 0.05). ARHGAP39 expression was positively associated with tumor stages and the mRNA expression in stage 3 was significantly higher than in stage 1 and stage 2 (Fig. 1E, *p* < 0.01). ARHGAP39 expression was correlated with patients’ age (Fig. 1F, *p *< 0.01). Considering the protein expression, higher ARHGAP39 level was found in luminal subtype than triple-negative subtype (Fig. 1G, *p* < 0.01). ARHGAP39 protein expression was correlated with cancer stage and patients’ age (Fig. 1H, I). Moreover, the relationship between ARHGAP39 expression and clinicopathological parameters from METABRIC data was shown in Supplementary Figure S1. ARHGAP39 was remarkably correlated with estrogen receptor (ER) (*p* = 0.0003), progesterone receptor (PR) (*p* = 0.0164) and HER2 status (*p* = 0.0458). Altogether, ARHGAP39 was upregulated in breast cancer and could be a treatment predictive biomarker during clinical decisions.

### ARHGAP39 prognosis analysis in breast cancer

The Kaplan–Meier curve analysis of the GSE1456 revealed that ARHGAP39 overexpression correlated with worse RFS (Fig. 1J, HR = 2.72, 95% CI = 1.27–5.83, *p* = 0.01), OS (Fig. 1K, HR = 2.44, 95% CI = 1.16–5.11, p = 0.02) and DSS (Fig. 1L, HR = 2.60, 95% CI = 1.08–6.26, *p* = 0.03) than a lower expression. The cut-point for RFS, OS and DSS was 0.65, 0.82 and 0.82, respectively. Furthermore, we performed targeted prognostic analyses for ARHGAP39 with all nodal status, ER status, and PR status patients with disease-free survival (DFS, *n* = 6470) and OS (*n* = 4577) using bc-GenExMiner v4.9. Tumors with higher levels of ARHGAP39 were linked to worse DFS (Supplementary Figure S2A, HR = 1.17, 95% CI = 1.08–1.27, *p* = 0.0001) and OS (Supplementary Figure S2B, HR = 1.16, 95% CI = 1.07–1.27, *p* = 0.0008). The cut-point for DFS and OS was 0.65. Similar results were found by analyzing the METABRIC data in Supplementary Figure S2, and the upregulated expression resulted in poor DFS (Supplementary Figure S2C, HR = 1.21, 95% CI = 1.08–1.35, *p* = 0.001) and OS (Supplementary Figure S2D, HR = 1.26, 95% CI = 1.12–1.41, *p* = 0.0001). The cut-point for DFS and OS was 0.55. Kaplan–Meier survival analysis based on TCGA database indicated that high expression of ARHGAP39 was associated with poor OS (Supplementary Figure S2E, *p* = 0.0014). Generally, ARHGAP39 was found to be significantly associated with worse survival outcomes.

### The impact of ARHGAP39 on cell proliferation, migration, and invasion

**Fig. 2:**
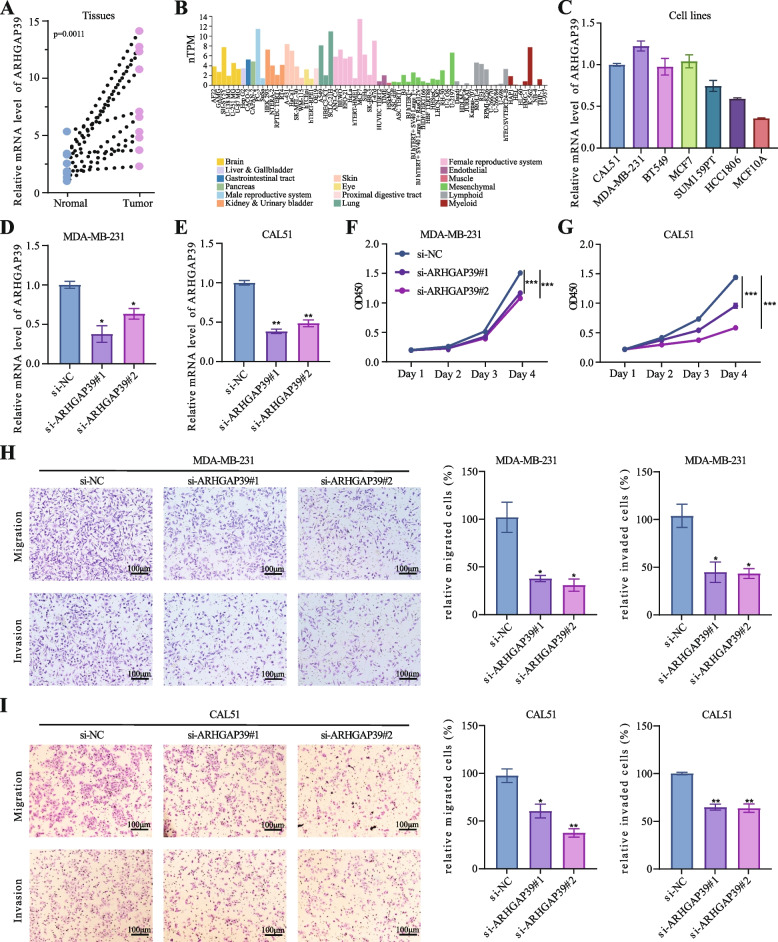
ARHGAP39 promotes breast cancer cell proliferation, migration, and invasion. **A** Difference in ARHGAP39 mRNA expression level between breast cancer tissues and adjacent normal tissues by qRT-PCR. **B** Difference in ARHGAP39 mRNA expression level in various cancer cell lines from HPA database. **C** Difference in ARHGAP39 mRNA expression between breast cancer cell lines and mammary epithelial cell line by qRT-PCR. **D**-**E** The transfection efficiency of ARHGAP39 siRNA in MDA-MB-231 and CAL51. **F**-**G** The CCK8 assay to detect the function of ARHGAP39 on cancer cell proliferation. **H**-**I** The transwell migration and invasion assays to detect the function of ARHGAP39 on cancer cell migrative and invasive capacity. *, *p* < 0.05; **, *p* < 0.01 by two-tailed Student’s t test

The expression and prognosis analysis indicated the oncogenic role of ARHGAP39 in breast cancer, and we determined its biological function in carcinogenesis. ARHGAP39 was particularly upregulated in breast cancer tissues (*n* = 8) compared with adjacent normal tissues by qRT-PCR analysis (Fig. 2A, *p* = 0.0011). Then, mRNA expression level of ARHGAP39 in human cell lines from HPA datasets were performed in Fig. 2B, and ARHGAP39 was upregulated in human breast cancer cells. Next, we assessed ARHGAP39 mRNA levels by RT-qPCR in CAL51, MDA-MB-231, BT549, MCF7, SUM159PT, HCC1806 and MCF10A cell lines. The results verified that ARHGAP39 was increased in cancer cells in comparison with MCF10A epithelial cell control (Fig. 2C). Under the condition of relative expression level, we knocked down ARHGAP39 by introducing two independent specific siRNAs in MDA-MB-231 and CAL51 breast cancer cells, followed by qRT-PCR analysis (Fig. 2D-E). CCK-8 assays revealed that knockdown of ARHGAP39 in MDA-MB-231 and CAL51 dramatically reduced cell proliferation (Fig. 2F-G). Ablation of KLHL29 dramatically inhibited the colony formation of MDA-MB-231 cells (Supplementary Figure S3A). Moreover, transwell assays showed that depletion of ARHGAP39 considerably reduced the migration and invasion ability of MDA-MB-231 and CAL51 cells (Fig. 2H-I). Wound healing experiments revealed that KLHL29 knockdown decreased the migratory potential of MDA-MB-231 (Supplementary Figure S3B). Considering the subcellular location, ARHGAP39 mainly localized in the cytosol, and additionally in the nucleoplasm and microtubules (Supplementary Figure S4) [35]. Together, we confirmed that ARHGAP39 could promote the proliferation, migration, and invasion of breast cancer.

### Co-expression network of ARHGAP39

**Fig. 3:**
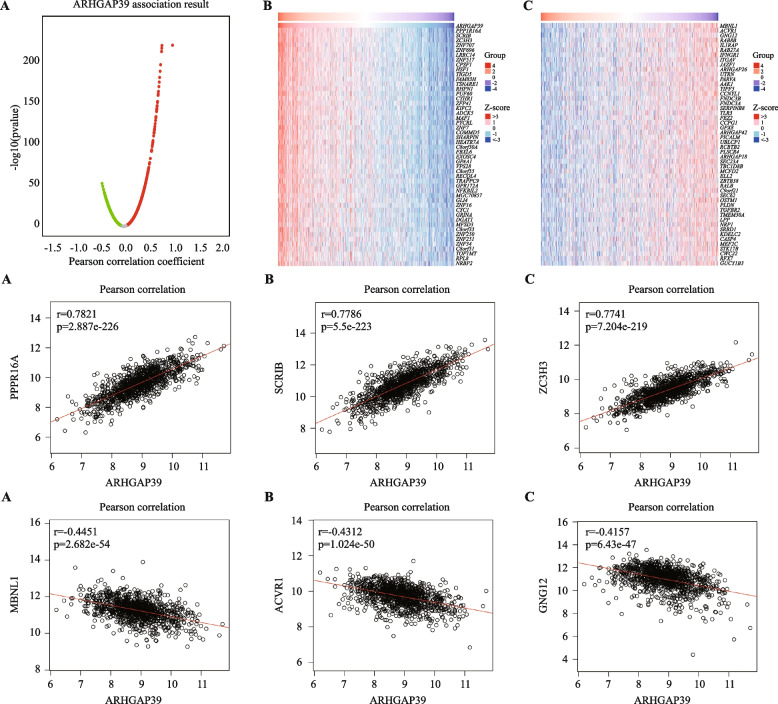
ARHGAP39 gene co-expression network in BRCA. **A** Volcano map showing the co-expressed genes associated with ARHGAP39 expression in breast cancer based on TCGA database. **B**-**C** Heat maps showing the top 50 co-expression genes positively and negatively correlated with ARHGAP39 in breast cancer. **D**-**E** Pearson correlation between ARHGAP39 expression with expression of PPP1R16A (**D**), SCRIB (**E**), ZC3H3 (**F**), MBNL1 (**G**), ACVR1 (**H**) and GNG12 (I)

We identified co-expressed genes of ARHGAP39 in the BRCA dataset of TCGA using the Linkedomics database. As illustrated in volcano plot (Fig. 3A), 5072 genes (red dots) showed positive correlation, and 6196 genes (green dots) showed negative correlation with ARHGAP39. Top 50 significant genes correlated with ARHGAP39 expression level were identified using heatmap (Fig. 3B, C). Our findings uncovered a widespread influence of ARHGAP39 on the transcriptome. The top 3 genes positively associated with ARHGAP39 were PPP1R16A (*r* = 0.7821, *p* = 2.287E-226), SCRIB (*r* = 0.7786, *p* = 5.5E-223), and ZC3H3 (*r* = 0.7741, *p* = 7.204E-219),and genes negatively correlated with ARHGAP39 were MBNL1 (*r* = -0.4451, *p* = 2.682E-54), ACVR1 (*r* = -0.4312, *p* = 1.024E-50), GNG12 (*r* = -0.4157, *p* = 6.43E-47). Subsequently, we evaluated the mRNA expression level of PPP1R16A, SCRIB, and ZC3H3 based on TCGA database, and protein expression level of PPP1R16A based on CPTAC database (Supplementary Figure S5), which might explain the underlying mechanism. Furthermore, the STRING database was employed to construct a PPI network of ARHGAP39. There were 21 edges and 11 nodes in the PPI network (PPI enrichment *p* value = 0.00237). The GO functional enrichments of ARHGAP39 and its interacting proteins were performed in Supplementary table S1. The significant GO terms enriched in BP were negative regulation of chemotaxis and negative regulation of chemokine-mediated signaling pathway.

### GO and KEGG pathway enrichment analysis

**Fig. 4:**
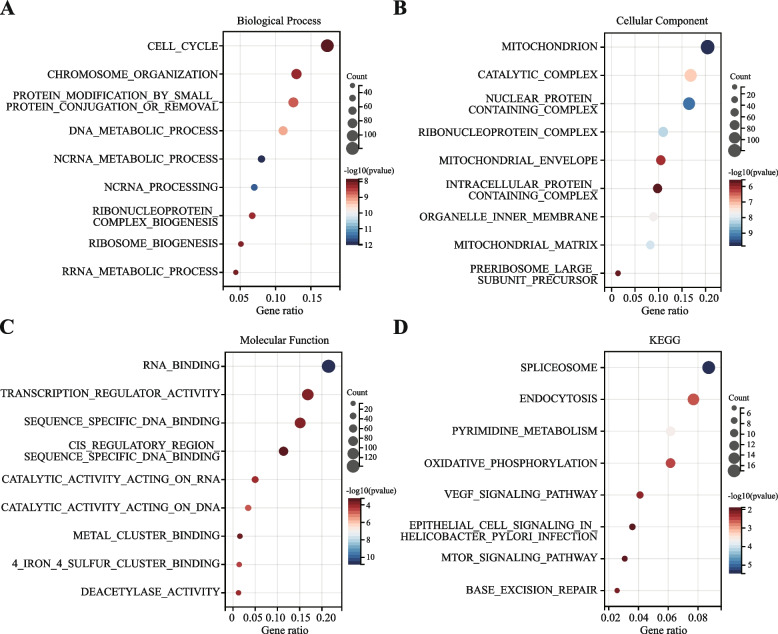
Enrichment analysis of GO and KEGG terms for ARHGAP39 co-expressed genes. **A**-**C** Enrichment analysis of GO terms. **A** Biological processes. **B** Cellular components. **C** Molecular functions. **D** KEGG pathways

We performed GO and KEGG pathway enrichment analyses of 1000 genes with strong correlation with ARHGAP39 in TCGA BRCA cohort by R package’s cluster Profiler and ranked by *p* value. The detailed enrichment terms in BP, CC, MF, and KEGG groups were summarized in Supplementary table S2-5. GO BP revealed enrichment in ncRNA metabolic process, ncRNA processing, DNA metabolic process, protein modification by small protein conjugation or removal, ribonucleoprotein complex biogenesis, chromosome organization, ribosome biogenesis, rRNA metabolic process, and cell cycle (Fig. 4A). CC showed enrichment in mitochondrion, nuclear protein containing complex, ribonucleoprotein complex, mitochondrial matrix, organelle inner membrane, catalytic complex, mitochondrial envelope, preribosome large subunit precursor, and intracellular protein containing complex (Fig. 4B). The enrichment terms in MF were RNA binding, catalytic activity acting on DNA, 4 iron, 4 sulfur cluster binding, catalytic activity acting on RNA, deacetylase activity, sequence specific DNA binding, transcription regulator activity, metal cluster binding, and cis regulatory region sequence specific DNA binding (Fig. 4C). KEGG pathway analysis indicated significant enrichment in spliceosome, pyrimidine metabolism, endocytosis, oxidative phosphorylation, VEGF signaling pathway, base excision repair, mTOR signaling pathway, and epithelial cell signaling in *Helicobacter Pylori* infection (Fig. 4D).

### Gene Set Enrichment Analysis

**Fig. 5:**
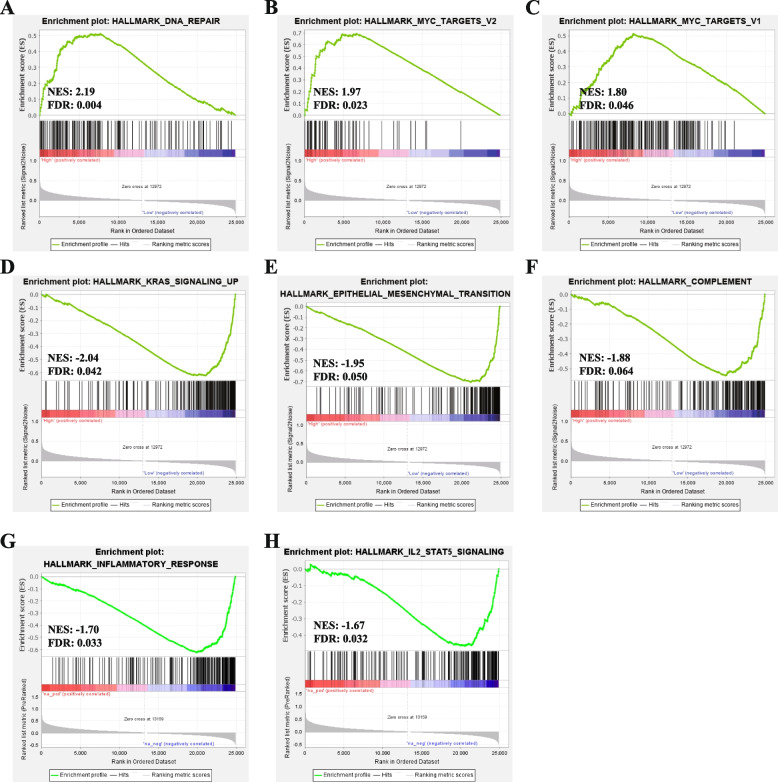
Gene set enrichment analysis. The enriched pathways included (**A**) DNA repair, (**B**) MYC targets V2, (**C**) MYC targets V1, (**D**) KRAS signaling up, (**E**) Epithelial mesenchymal transition, (**F**) Complement, (**G**) Inflammatory response, (**H**) IL2/STAT5 signaling pathway

We conducted GSEA analysis to identify important pathways to characterize the potential biological mechanisms of ARHGAP39 in breast cancer. Detailed hallmark pathways enrichment analysis information was shown in Supplementary Table S6. The crucial pathways included DNA repair (Fig. 5A), MYC targets V2 (Fig. 5B), MYC targets V1(Fig. 5C), KRAS signaling up (Fig. 5D), epithelial mesenchymal transition (Fig. 5E), and complement (Fig. 5F), inflammatory response (Fig. 5G) and IL2/STAT5 signaling pathway (Fig. 5H). Collectively, ARHGAP39 might influence immune infiltration, and the role of ARHGAP39 in tumor immunology of breast cancer should be comprehensively analyzed.

### Correlation between ARHGAP39 and immune cell infiltration

**Fig. 6:**
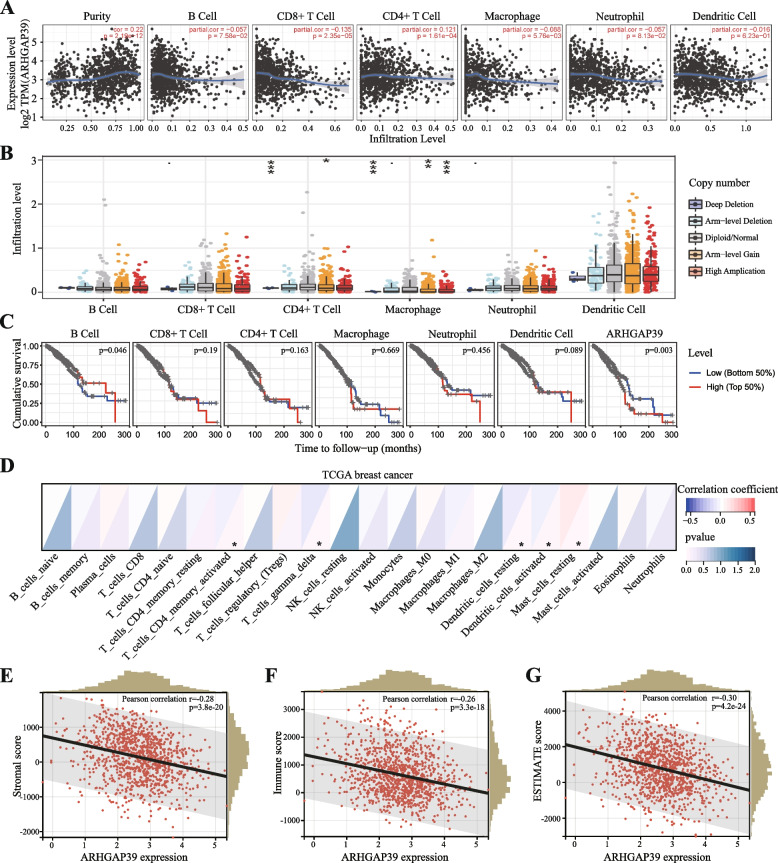
Correlation between ARHGAP39 expression and tumor immune infiltrating cells and immune score. **A** The correlation between ARHGAP39 expression and immune infiltrating cells. **B** The correlation between ARHGAP39 CNV and immune infiltrating cells. **C** Kaplan–Meier plotter of immune infiltration and ARHGAP39 expression levels in BRCA. **D** The correlation of ARHGAP39 expression with 22 tumor immune cell infiltration using CIBERSORT analysis. **E**–**G** The correlation of ARHGAP39 expression with stromal, immune, and estimate scores. *, *p* < 0.05; **, *p* < 0.01; ***, *p* < 0.001

The TIMER database was generated to analyze the correlation between ARHGAP39 expression and immune infiltrating cells. ARHGAP39 was negatively correlated with infiltration level of CD8 + T cell (*r* = -0.135, *p* = 2.35E-05), and macrophage (*r* = -0.088, *p* = 5.76E-03), while positively associated with CD4 + T cell (*r* = 0.121, *p* = 1.61E-04). No significant correlation was found in B cell, neutrophil, and dendritic cell (Fig. 6A). The relationship between ARHGAP39 and cell markers of macrophage in TIMER database was performed in Table 1 and Supplementary Figure S6. The expression of gene markers of M1 macrophages (ARG2 and PTGS2), M2 macrophage (VSIG4), and TAM (CCL2 and CD86) were significantly negatively linked to ARHGAP39 expression. Next, we further detected the relationship between co-expressed gene of ARHGAP39 and immune cell infiltration. PPP1R16A expression was negatively associated with B cell, CD8 + T cell, macrophage, and neutrophil, while was positively associated with CD4 + T cell (Supplementary Figure S5D). SCRIB and ZC3H3 expression had a negative correlation with CD8 + T cell and macrophage, while had a positive correlation with CD4 + T cell (Supplementary Figure S5E-F). Taken together, we demonstrated that ARHGAP39 and its co-expressed genes were involved in the immune-related pathways by affecting immune infiltrating cells, especially CD8 + T cell, macrophage, and CD4 + T cell.

**Table 1:** Correlation analysis between ARHGAP39 expression and immune cell marker gene in TIMER

Description	Gene markers	None	*P*-value	partial.cor	partial.p
M1 Macrophage	NOS2	-0.0269392	0.37206072	-0.0133137	0.67503935
	ARG2	-0.1006587	0.00082827	-0.1262338	**6.58E-05**
	PTGS2	-0.1575282	1.51E-07	-0.1003232	**0.00154031**
M2 Macrophage	CD163	-0.0677636	0.02460845	0.00595461	0.85126785
	MRC1	-0.1146002	0.00013948	-0.0198841	0.53120126
	VSIG4	-0.134141	8.04E-06	-0.0648804	**0.04084348**
	MS4A4A	-0.1368398	5.24E-06	-0.0496402	0.11780828
	CD209	-0.1061413	0.00042146	-0.0205705	0.51711815
TAM	CCL2	-0.1773776	3.16E-09	-0.1105743	**0.00047854**
	CD86	-0.1588213	1.19E-07	-0.0785375	**0.01325571**
	CD68	-0.124419	3.50E-05	-0.0505953	0.11089772
	IL10	-0.0610386	0.04296942	0.01529698	0.63001852

We demonstrated that ARHGAP39 CNV was closely associated with CD4 + T cell and macrophages’ infiltration degree (Fig. 6B). Kaplan–Meier curve analysis was applied to explore the correlation between survival and the abundance of ARHGAP39 expression and immune cells. We found that B cell infiltration (*P* = 0.046) was significantly associated with the prognosis of BRCA (Fig. 6C). CIBERSORT analysis revealed significant correlation between ARHGAP39 and activated memory T CD4 + cell, gamma delta T cell, resting dendritic cells, activated dendritic cells, and resting mast cell (Fig. 6D). Regarding the immune score analysis, the expression of ARHGAP39 displayed a negative correlation with stromal score (Fig. 6E, *r* = -0.28, *p* = 3.8E-20), immune score (Fig. 6F, *r *= -0.26, *p* = 3.3E-18), and ESTIMATE score (Fig. 6G, *r* = -0.30, *p* = 4.2E-24).

### Correlation between ARHGAP39 and immunomodulators

**Fig. 7:**
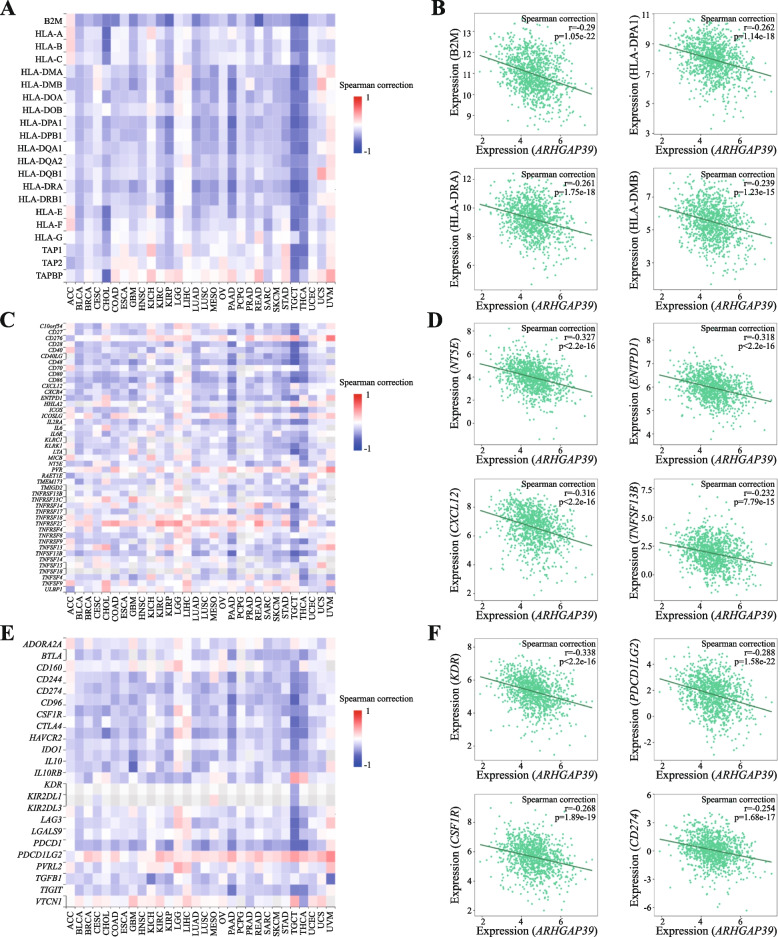
Relationship between ARHGAP39 expression and the abundance of immunomodulators in BRCA using TISIDB. **A**-**B** The correlation between ARHGAP39 expression and the abundance of MHC molecules and top 4 MHC molecules showing the greatest correlation with ARHGAP39 expression. **B** The correlation between ARHGAP39 expression and the abundance of immunostimulators and top 4 immunostimulators showing the greatest correlation with ARHGAP39 expression. **C** The correlation between ARHGAP39 expression and the abundance of immunoinhibitors and top 4 immunoinhibitors showing the greatest correlation with ARHGAP39 expression

To further illustrate novel function of ARHGAP39, we used the TISIDB database to evaluate the relationship between ARHGAP39 expression and the abundance of immunomodulators. Figure 7A-B showed that ARHGAP39 had negative correlation with numerous MHC molecules, especially with B2M (Spearman: rho = -0.29, *p* = 1.05E − 22), HLA-DPA1 (Spearman: rho = -0.262, *p* = 1.14E − 18), HLA-DRA (Spearman: rho = -0.261, *p* = 1.75E-18), and HLA-DMB (Spearman: rho = -0.239, *p* = 1.23e − 15). In addition, ARHGAP39 expression was negatively correlated with multiple immune stimulators (Fig. 7C-D), and four immunostimulators with strong correlation were NT5E (Spearman: rho = -0.327, *p* < 2.2E − 16), ENTPD1 (Spearman: rho = -0.318, *p* < 2.2E − 16), CXCL12 (Spearman: rho = -0.316, *p* < 2.2E − 16), and TNFSF13B (Spearman: rho = -0.232, *p* = 7.79E − 15). Four immunoinhibitors (Fig. 7E-F) with strong correlation were KDR (Spearman: rho = -0.338, *p* < 2.2E − 16), PDCD1LG2 (Spearman: rho = -0.288, *p* = 1.58E − 22), CSF1R (Spearman: rho = -0.268, *p* < 1.89E − 19) and CD274 (Spearman: rho = -0.254, *p* = 1.68E − 17). The above results indicated that ARHGAP39 might involve in immune response through the immunomodulators.

## Discussion

ARHGAP39, a member of the RhoGAP group, is also regarded as preoptic regulatory factor-2 (Porf-2) or Vilse, and is frequently involved in neurogenesis and neurodevelopment [36]. However, there is still a lack of evidence for the role of ARHGAP39 in breast cancer. Our study sheds light on understanding the underlying mechanisms of ARHGAP39 in tumor immunology and represents the diagnostic and therapeutic target for tailored therapy in breast cancer. First, we estimated the expression level and prognostic value of ARHGAP39 in breast cancer. Analysis of transcriptomes of breast cancer in TCGA database demonstrated higher ARHGAP39 expression in tumor tissues than normal tissues, and our qRT-PCR results reached a consistent conclusion. According to analysis of CPTAC database, higher ARHGAP39 protein expression was found in breast cancer tissues than that in normal tissues. Additionally, the differential expression level of ARHGAP39 was explored in biological subtypes, tumor grade, ER status, PR status, and HER2 status. Taken together, ARHGAP39 was identified as an upregulated biomarker which was significantly associated with advanced clinicopathological factors in breast cancer. Survival analysis indicated that overexpression of ARHGAP39 was correlated with poor prognosis, suggesting its potential prognostic role. We conducted in vitro experiments including cell viability assays and transwell assays to confirm the biological function of ARHGAP39, and the results confirmed that ARHGAP39 could foster breast cancer cell proliferation, migration, and invasion.

Current studies on the role of ARHGAP39 mainly focused on its effect on filopodia formation and endothelial cell migration [37], ganglion and axon tracking [14], neuronal cell proliferation and apoptosis [38], dendritic spine morphology [39], neurodevelopment and learning and memory [40, 41]. The potential biological roles of ARHGAP39 in oncogenesis were less studied. Co-expressed genes could play cooperative and complementary roles in the biological processes, thus, we generated co-expression network and conducted enrichment analysis. The GO enrichment analysis was conducted to predict the functions of the top 1000 genes positively associated with ARHGAP39, and the enrichment terms in BP, CC, and MF were ncRNA metabolic process, mitochondrion, and RNA binding. KEGG pathway analysis revealed that ARHGAP39 was correlated with spliceosome, pyrimidine metabolism, endocytosis, oxidative phosphorylation, VEGF signaling pathway, base excision repair and mTOR signaling pathway, which were proven to be crucial pathways associated with the development and progression of breast cancer.

ARHGAP39 showed a strong positive correlation with PPP1R16A, SCRIB, and ZC3H3. PPP1R16A, a protein coding gene, was reported to be associated with ovarian clear cell adenocarcinoma [42]. Our co-expression network indicated strong correlation between PPP1R16A and ARHGAP39, moreover, the PPI network showed the association. SCRIB, encoding the Scribble protein, localizes to cell–cell junctions and mediates the establishment of epithelial cell polarity, was considered as a regulator of tumor development and metastasis [43–45]. SCRIB can be act as oncogene or tumor suppressing gene in different tumors, while the biological function of SCRIB in breast cancer was shown to promote mammary tumorigenesis [46–48]. ZC3H3, methylated gene, was regarded to be involved in the regulation of mRNA polyadenylation and can act as risk indicator for predicting prognosis in bladder cancer and adrenocortical carcinoma [49, 50].

ARHGAP39 showed a strong negative correlation with MBNL1, ACVR1, and GNG12. MBNL1, a tissue-specific RNA metabolism regulator, was an important regulator of tumor metastasis and growth. High MBNL1 expression level in human breast tumors was found to be associated with reduced metastatic relapse likelihood and survival and promote tumor progression [51, 52]. MBNL1 could act as major regulator in monocyte-to-macrophage differentiation and activation, and regulate immune infiltration [53]. ACVR1, member of TGF-beta superfamily of structurally related signaling proteins, was linked to cell stemness, tumorigenicity, and immune microenvironment remodeling [54, 55]. GNG12 is a risk factor for several cancers, and a possible target for immunotherapy [56]. GNG12 is participated in the activation of the NF-kB signal, supporting the evasion of cancer immunity and in turn activating cancer proliferation, and angiogenesis [57]. The existing research results of the co-expressed gene could partially explain that ARHGAP39 as a prognostic biomarker influence immune infiltration in breast cancer.

The acquisition and maintenance of hallmarks of cancer including sustaining proliferative signaling, resisting cell death, activating invasion and metastasis, reprogramming cellular metabolism, and avoiding immune destruction are intrinsically correlated with TME [58]. TME, a crucial mediator of cancer progression, has attracted increasing researches and clinical interest in extending therapeutic intervention and exploring new approaches for tumors [59–61]. The composition and infiltration density of immune cells in TME profoundly influence tumor onset and development [62]. At present, we found an essential role of ARHGAP39 in breast cancer immunity. The GSEA analysis demonstrated that ARHGAP39 showed a negative correlation with immune-related pathways, especially the complement pathway, inflammatory response and IL2/STAT5 signaling pathway. In line with the enrichment analysis of ARHGAP family members, immune-related TGF-β, TNF- α, IL-2/STAT5, IL-6/JAK/STAT3, and the inflammatory response pathway were in relation with tumorigenesis.

Recent research has demonstrated that the immune system influences tumor development and the characteristics of immune cell infiltration were correlated with the immune therapeutic effect and clinical efficacy [63, 64]. The crosstalk between cancer cells and tumor associated immune cells might possess tumor-promoting and tumor-antagonizing effects [6, 65]. In our study, we found that ARHGAP39 was negatively associated with infiltrating levels of CD8 + T cell and macrophage, and positively associated with CD4 + T cell by using TIMER database. Its co-expressed genes were also involved in the immune-related pathways by affecting immune infiltrating cells, especially CD8 + T cell, macrophage, and CD4 + T cell. Moreover, gene markers of M1 macrophages such as ARG2 and PTGS2, M2 macrophage markers such as VSIG4, and TAM markers such as CCL2 and CD86 showed significant negative correlations. Tumor-associated macrophages exhibit crucial functions in facilitating biological pathologic processes of breast cancer cell and prospect therapeutic strategies for opposing tumor progression [66]. No significant correlation was found in B cell, neutrophil, and dendritic cell. These correlations could be indicative of a potential mechanism where ARHGAP39 regulates T cell functions and macrophage functions in breast cancer.

The CIBERSORT algorithm was supplementarily applied to quantify the proportion of 22 immune cell types, and we found that activated memory CD4 + T cell, gamma delta T cell, resting dendritic cells, activated dendritic cells were negatively correlated with ARHGAP39 level and resting mast cell were positively correlated with ARHGAP39 level. The ESTIMATE algorithm was used to predict tumor purity, the expression of ARHGAP39 showed significant negative correlation with immune score, stromal score, and ESTIMATE score. To further identify the role of ARHGAP39 in TME, the correlation of ARHGAP39 with immunomodulators was analyzed using the TISIDB database. The expression of ARHGAP39 were significantly negatively correlated with immunoinhibitors, immunostimulators and MHC molecules. Together these findings suggested that ARHGAP39 was indeed a determinant factor of immune infiltration and immunomodulators. The mechanism of ARHGAP39 in immune infiltration needs to be further explored.

In conclusion, our study provided a comprehensive profiling of ARHGAP39 in breast cancer which could strengthen our understanding of the molecular mechanisms of breast cancer and aid in biomarker discovery. We confirmed that ARHGAP39 was upregulated in breast cancer and its expression level was correlated with poor prognosis and advanced clinical characteristics. Furthermore, ARHGAP39 potentially regulated the immune-related pathways, and affected the tumor-infiltrating immune cells, thus providing opportunities for the development of novel immunotherapies for breast cancer treatment.

## Supplementary Information

**Additional file 1:** **Figure S1.**Relationship between ARHGAP39 expression and clinicopathological parameters. **Figure S2.** Kaplan-Meier curve ofARHGAP39 in breast cancer. **Figure S3.**ARHGAP39 promotes breast cancer cell colony formation and wound healingability. **Figure S4.** Subcellularlocation of ARHGAP39. **Figure S5.**Relationship between ARHGAP39 co-expressed genes and the immune cellinfiltration level. **Figure S6.**Relationship between ARHGAP39 and immune cell marker gene of macrophage inTIMER. **Figure S7.** The protein-protein interactions network of ARHGAP39.

**Additional file 2:** **Supplementary Table S1.** The GO functionalenrichments of ARHGAP39 and its interacting proteins. **Supplementary Table S2.** Biological Processes. **Supplementary Table S3.** Cellular Components. **Supplementary Table S4.** Molecular Functions. **Supplementary Table S5.** KEGG Pathways. **Supplementary Table S6.** GSEA Analysis.

## Data Availability

The datasets presented in this study can be found in online repositories including: The UCSC XENA (https://xena.ucsc.edu/). Clinical Proteomic Tumor Analysis Consortium (CPTAC) database (https://proteomics.cancer.gov/programs/cptac/). ULCAN online tools (http://ualcan.path.uab.edu/). STRING database (https://string-db.org/cgi/input.pl). Human Protein Atlas (HPA) database (http://www.proteinatlas.org/). LinkedOmics database (http://www.linkedomics.org). TIMER database (https://cistrome.shinyapps.io/timer/). TISIDB database (http://cis.hku.hk/TISIDB/index.php).
