# Supplementary material for: ARHGAP39 is a prognostic biomarker involved in immune infiltration in breast cancer
Source: BMC Cancer. 2023 May 15;23:440. doi: 10.1186/s12885-023-10904-4 (PMC10184379; doi:10.1186/s12885-023-10904-4)
Supplement: Supplementary file 1 — Additional file 1: Figure S1.Relationship between ARHGAP39 expression and clinicopathological parameters. Figure S2. Kaplan-Meier curve ofARHGAP39 in breast cancer. Figure S3.ARHGAP39 promotes breast cancer cell colony formation and wound healingability. Figure S4. Subcellularlocation of ARHGAP39. Figure S5.Relationship between ARHGAP39 co-expressed genes and the immune cellinfiltration level. Figure S6.Relationship between ARHGAP39 and immune cell marker gene of macrophage inTIMER. Figure S7. The protein-protein interactions network of ARHGAP39. [file 12885_2023_10904_MOESM1_ESM.pdf]

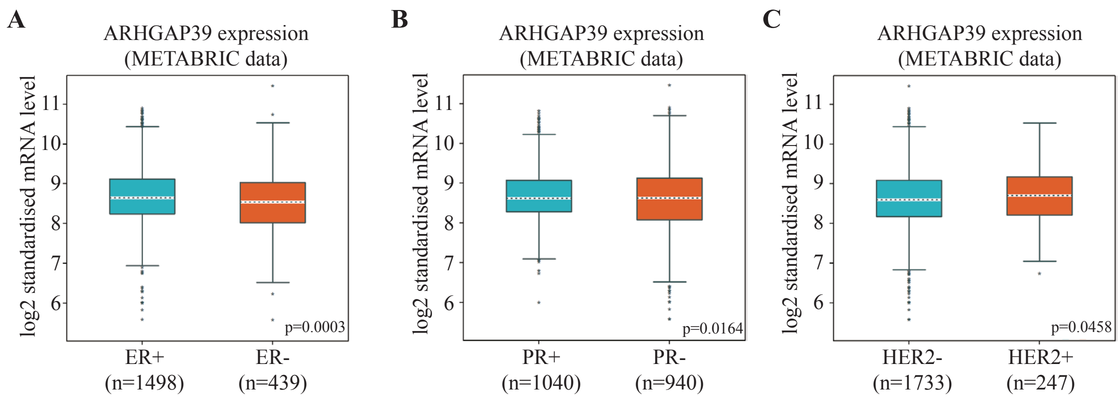

Figure S1. Relationship between ARHGAP39 expression and clinicopathological parameters.

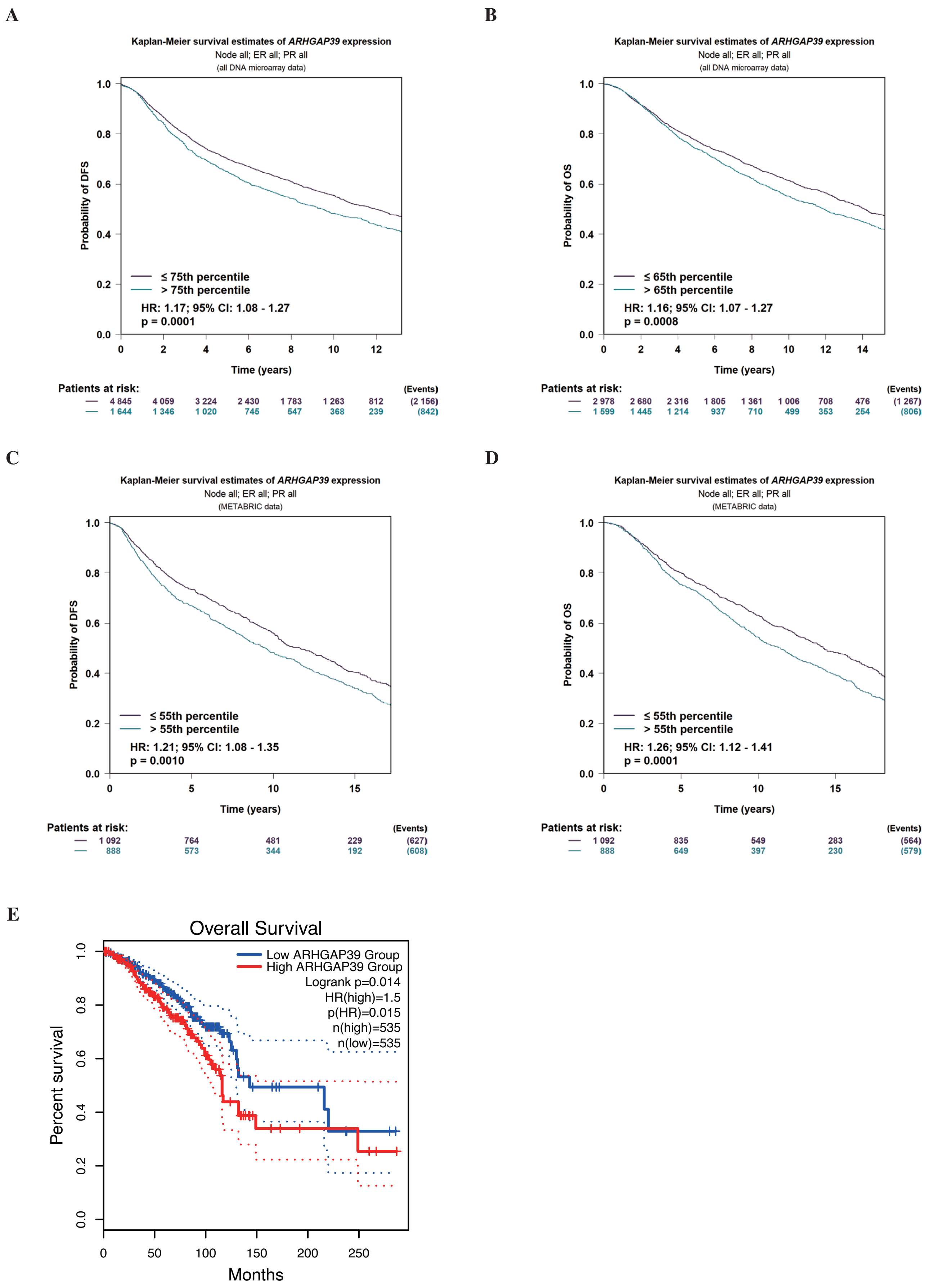

Figure S2. Kaplan-Meier curve of ARHGAP39 in breast cancer.

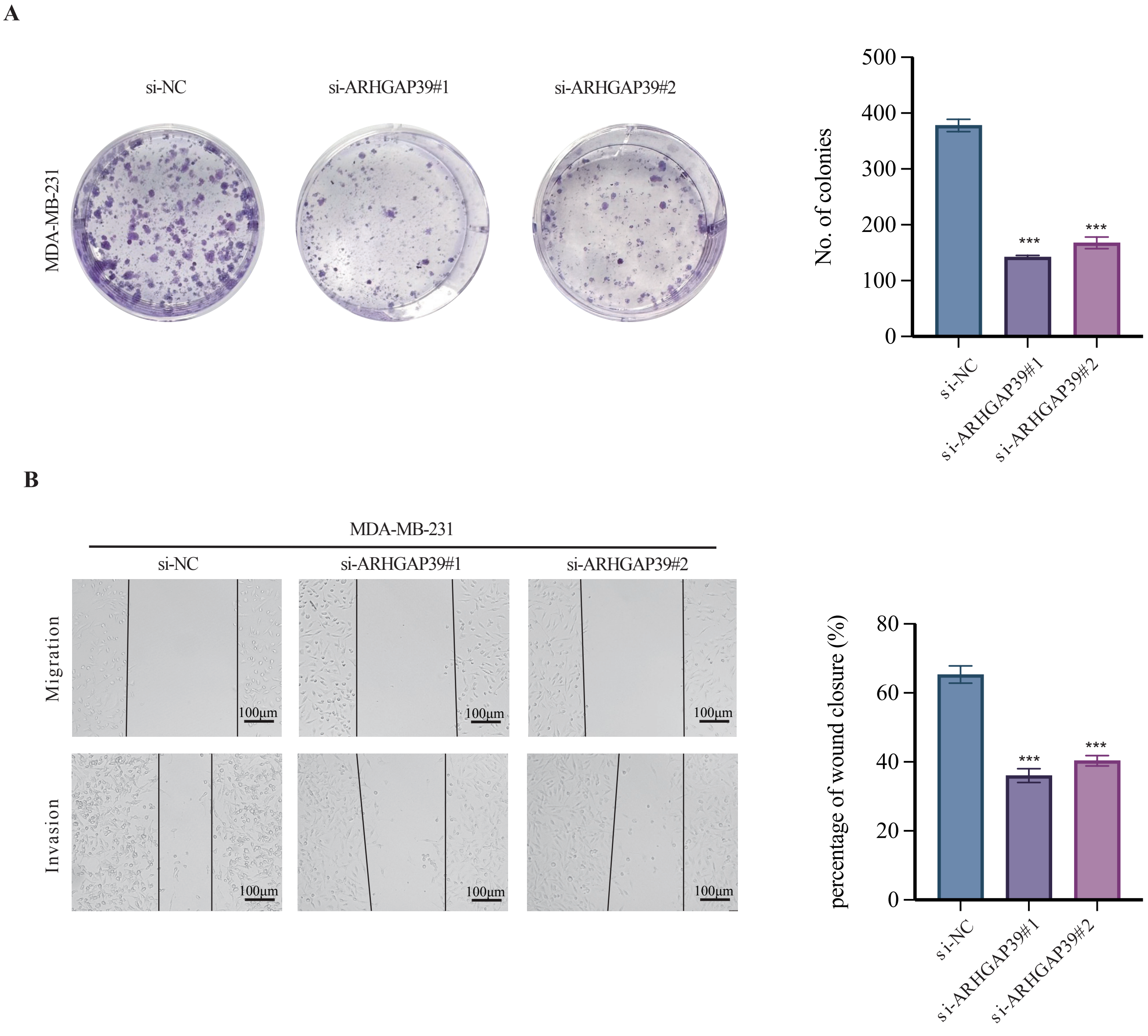

Figure S3. ARHGAP39 promotes breast cancer cell colony formation and wound healing ability.

A

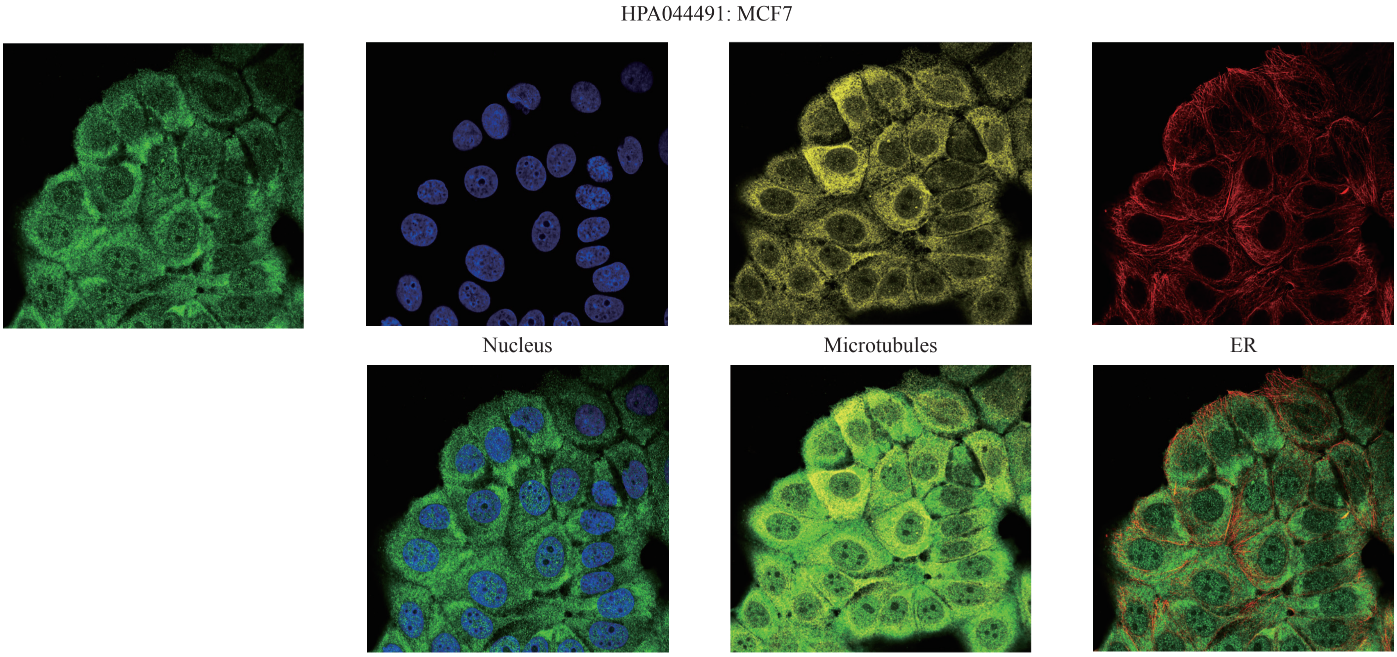

B

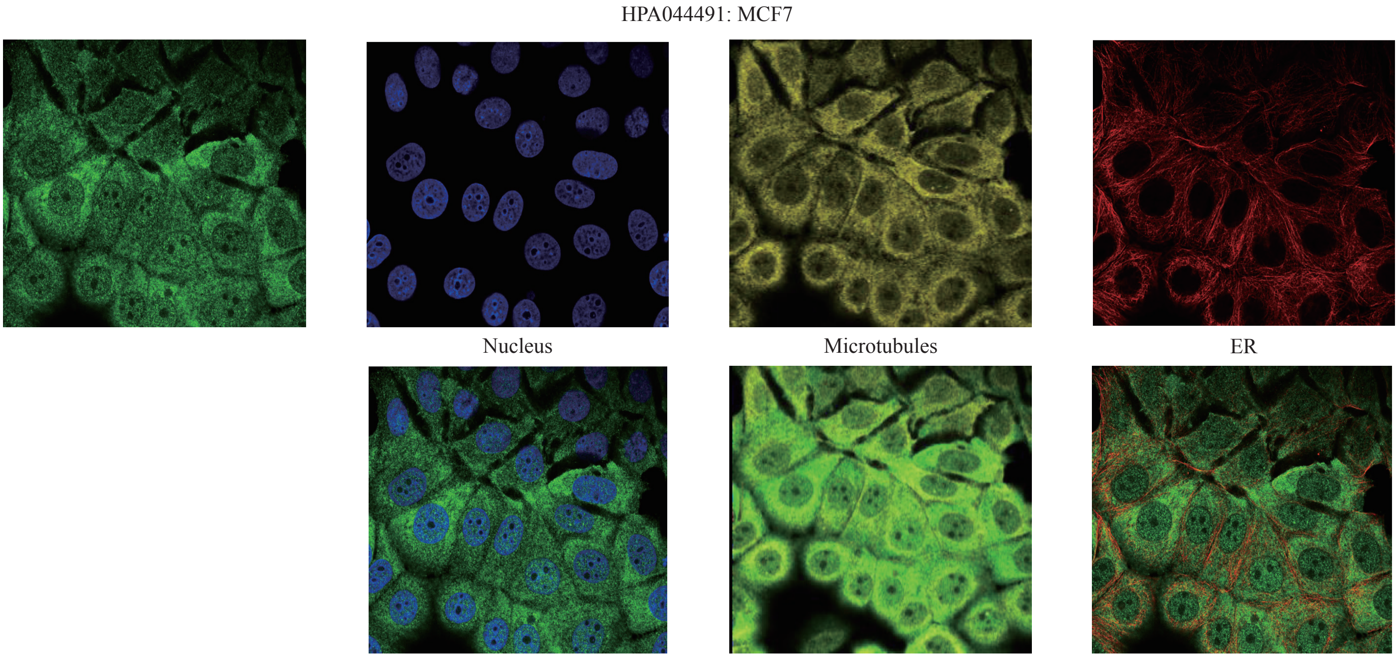

Figure S4. Subcellular location of ARHGAP39.

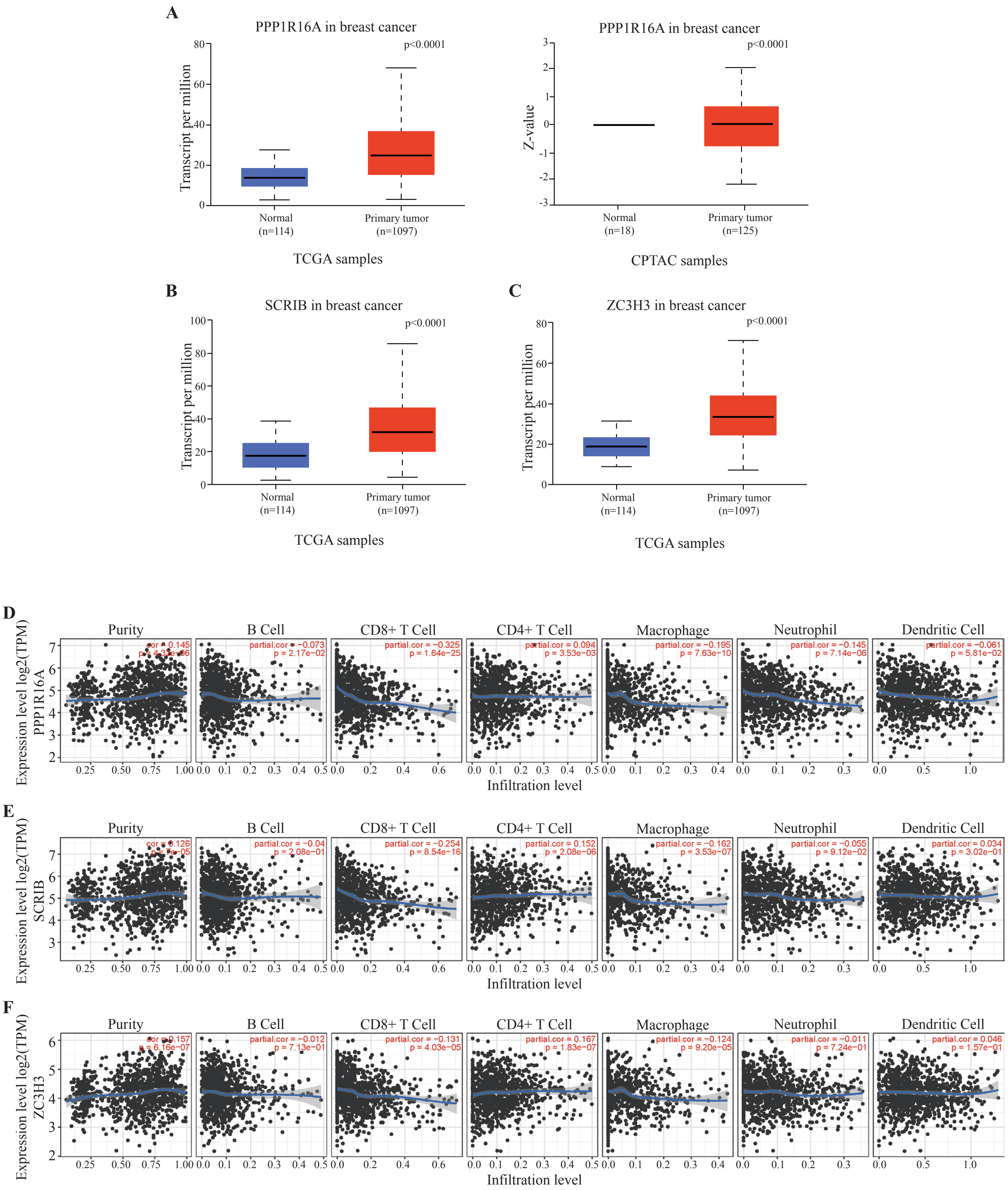

Figure S5. Relationship between ARHGAP39 co-expressed genes and the immune cell infiltration level.

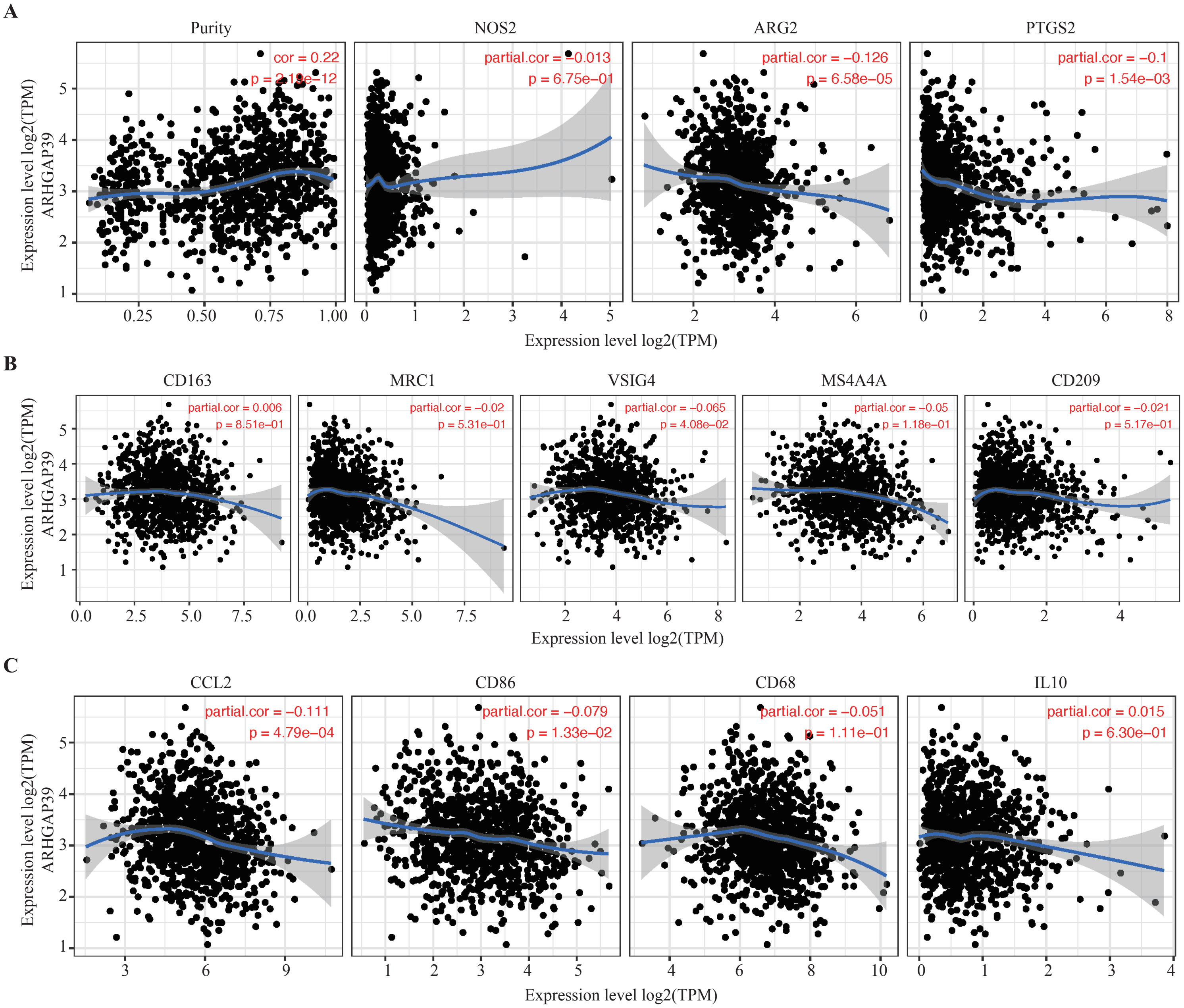

Figure S6. Relationship between ARHGAP39 and immune cell marker gene of macrophage in TIMER.

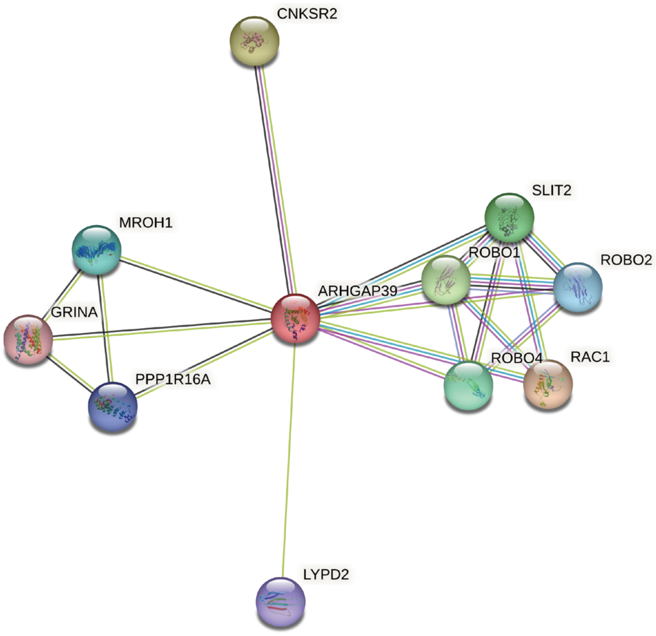

Figure S7. The protein-protein interactions network of ARHGAP39.
